# Detection of Bird Nests during Mechanical Weeding by Incremental Background Modeling and Visual Saliency

**DOI:** 10.3390/s150305096

**Published:** 2015-03-02

**Authors:** Kim Arild Steen, Ole Roland Therkildsen, Ole Green, Henrik Karstoft

**Affiliations:** 1 Department of Engineering, Aarhus University, Finlandsgade 22, 8200 Aarhus N, Denmark; E-Mail: hka@eng.au.dk; 2 Department of Bioscience, Aarhus University, Grenåvej 14, 8410 Rønde, Denmark; E-Mail: oth@dmu.dk; 3 Kongskilde Industries, Strategic Development, Niels Pedersens Allé 2, 8830 Tjele, Denmark; E-Mail: olg@kongskilde.com

**Keywords:** background modeling, visual saliency, obstacle detection, mechanical weeding, computer vision

## Abstract

Mechanical weeding is an important tool in organic farming. However, the use of mechanical weeding in conventional agriculture is increasing, due to public demands to lower the use of pesticides and an increased number of pesticide-resistant weeds. Ground nesting birds are highly susceptible to farming operations, like mechanical weeding, which may destroy the nests and reduce the survival of chicks and incubating females. This problem has limited focus within agricultural engineering. However, when the number of machines increases, destruction of nests will have an impact on various species. It is therefore necessary to explore and develop new technology in order to avoid these negative ethical consequences. This paper presents a vision-based approach to automated ground nest detection. The algorithm is based on the fusion of visual saliency, which mimics human attention, and incremental background modeling, which enables foreground detection with moving cameras. The algorithm achieves a good detection rate, as it detects 28 of 30 nests at an average distance of 3.8 m, with a true positive rate of 0.75.

## 1. Introduction

In organic farming, weed control is based on mechanical solutions, but increased focus on decreasing the use of pesticides in the conventional sector and an increased number of pesticide-resistant weeds has created renewed interest in mechanical weeding. At the same time, the precision and capacity of mechanical weed-control solutions have grown considerably. One therefore expects a significant growth in the use of semi-autonomous agricultural machinery in the coming years.

Agricultural fields provide nesting habitat to a wide range of farmland bird populations, such as skylark (*Alauda arvensis*), corn bunting (*Emberiza calandra*) and grey partridge (*Perdix perdix*), many of which have declined in recent years [1]. However, ground nesting birds are highly susceptible to farming operations, which may destroy the nests and reduce the survival of chicks and incubating females. Therefore, in combination with other factors, e.g., higher predation levels, breeding in agricultural fields, such as intensively-managed hayfields and row crops, is often characterized by relatively low reproductive success, and it is suggested that such habitats probably represent population “sinks” [2,3]. Indeed, in [4], it was shown that 50%–100% of sky lark nests were destroyed by weeding row crops.

Since wildlife-friendly farming often results in lower efficiency, attempts have been made to develop automatic systems capable of detecting wild animals in the crop [5,6]. However, these systems have focused on larger species, *i.e.*, fawns of roe deer (*Capreolus capreolus*) and leverets of brown hare (*Lepus europaeus*), which are more easily detected and may be moved to safety upon detection in the field. It is therefore necessary to explore and develop new technological solutions to ensure ethical and effective crop production.

Monocular vision and stereo vision are commonly used in obstacle detection [7–10]. Here, obstacles are found via 3D reconstruction, either via relative motion (monocular vision) or disparity maps (stereo vision). For ground-based vehicles, obstacles are usually defined as objects that extend above the ground plane [7–12]. This approach is not suited for the detection of bird nests in an agricultural setting, since bird nests are located on the ground and may be smaller than the crops in height. Hence, spatial information alone is not useful for recognizing these regions in the image as an obstacle. Other monocular-based obstacle detection algorithms include appearance-based models [9,13], thresholding in the HSI (hue, saturation and intensity) color space [10] and relative size [14].

In computer vision, visual saliency algorithms attempt to mimic human visual attention [15,16]. Salient objects in an image are defined as the part of an image that catches our attention or objects that are of most interest [15]. Visual saliency has been applied to both monocular- [17] and stereo-based obstacle detection [18,19]; however, here, the visual saliency algorithm has been used as part of the 3D reconstruction of the scene in order to detect obstacles that extend above the ground plane. Visual saliency could also be used in earlier stages of the image processing chain, to detect regions that could contain obstacles, regardless of the geometrical properties. This is part of the proposed algorithm in this paper.

Background subtraction is widely used in surveillance tasks to detect objects of interest. Here, the camera is usually capturing a more or less static scene [20], although algorithms for highly dynamic scenes have been proposed [21]. The output from background subtraction is the regions of an image that lie outside a defined background model. In static setups, this model can be derived from median filtering over time [20] or a Gaussian mixture model, which models the expected intensity or RGB values of a specific pixel in an image [22]. These approaches are not suitable for moving cameras, since the assumptions for static setups are violated. In [9,13], a background model for moving cameras is presented. Here, the model is based on background appearance and found via histogram methods. In this paper, we base the background model on background appearance and introduce an incremental update scheme based on visual saliency. The model exploits the homogeneity of row crop fields.

In this paper, we present an algorithm for nest detection during mechanical weeding. We treat these nests as obstacles that should be avoided. However, these obstacles are placed on the ground and potentially smaller in height than the crops in the field. Hence, detection based on spatial information is not suitable. Instead, we propose an algorithm driven by visual attention. Here, both visual saliency and incremental background modeling are fused to enable the detection of ground-lying nests while driving towards them.

## 2. Materials and Methods

### 2.1. Data Collection

**Figure 1.**
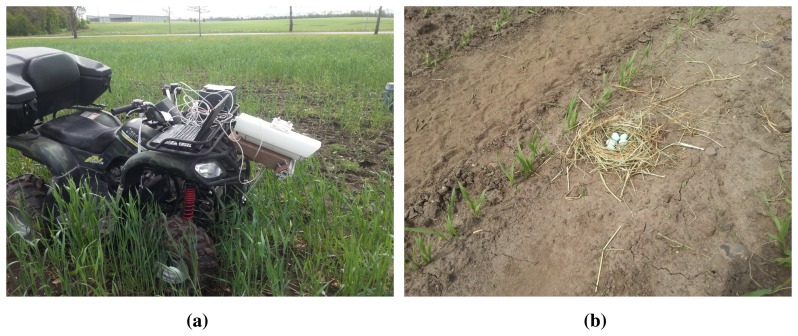
Photos from data collection. (**a**) Thermo-visual setup mounted on an all-terrain vehicle; (**b**) man-made skylark nest with heated plastic eggs.

A thermal camera (FLIR A320) with a resolution of 380 × 240 pixels and a field of view of 45° × 34° (H × V) and an RGB camera (Basler acA1600-20gc) with a resolution of 1624 × 1234 pixels and a field of view of 43° × 34° were mounted next to each other on the front of an all-terrain vehicle. The cameras were placed approximately 80 cm above the field and tilted 20°, to mimic the placement of a similar camera setup on mechanical weeding machinery (Figure 1a). The cameras recorded uncompressed images at a frame rate of approximately 5 frames per second (limited by the thermal camera). To compensate for the low frame rate, the vehicle was driven at speeds of around 4–5 km/h.

Man-made nests with 3–5 heated plastic eggs were placed on the ground in various crops (Figure 1b). The eggs were heated to a temperature of approximately 35 °C (when they were placed in the nest) to resemble the temperature during incubation. The recordings took place 22 May 2014, and the weather was sunny with an ambient temperature of around 25–26 °C.

**Figure 2.**
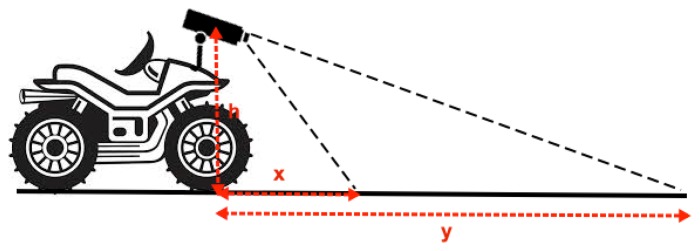
Geometrical properties of the setup for recording.

The camera placement on the ATV gives an imaging range of 1–15 m in front of the vehicle. This is illustrated in Figure 2, where h denotes the height of the camera, which was 0.8 m above the ground plane, and x and y denote the range, which are approximately x = 1 m and y = 15 m for both cameras.

**Figure 3.**
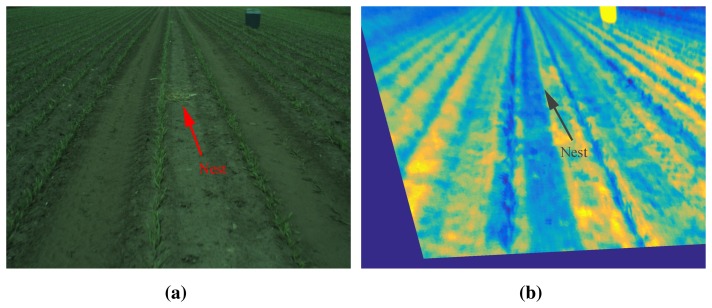
Images captured by the RGB and thermal camera. (**a**) Visual image of the field, where the nest is placed in the middle of the image; (**b**) the same scenario in the thermal domain (here, it is impossible to see the nest).

A single frame from the recordings is shown in Figure 3 (visual and thermal). A man-made nest is shown in the center of both images. However, in the thermal image, the nest is impossible to locate visually. Two factors make it almost impossible to detect the nest using the thermal camera. First, the weather conditions have a great influence on the quality of the thermal image (with respect to detection), as the surrounding soil is being heated by the Sun, making the difference in temperature between field and eggs very small. Secondly, the eggs are so small that the thermal camera does not easily detect them. Hence, we chose to omit thermal images in the nest detection algorithm presented in this paper.

A total of 15 recordings, containing 68–134 frames, corresponding to driving distances of 9–19 m, were made. The recordings all contain 2 bird nests, placed on the ground approximately 7 m apart. The cooling box seen in the images was used for storage of the eggs and has been manually removed in the analysis of nest detection performance.

### 2.2. Visual Saliency

Visual saliency algorithms attempt to mimic human visual attention [15,16]. Salient objects in an image are defined as the part of an image that catches our attention or objects that are of most interest [15]. In the literature, different approaches to calculating saliency have been presented. Among the most popular is the version presented in [16]. Here, nine spatial scales are created using dyadic Gaussian pyramids and used to calculate feature maps for both intensity, color and orientation. The features are computed based on the center-surround features, which mimic the visual receptive fields. Center-surround features are calculated as the difference between fine and coarse scales in the dyadic Gaussian pyramid framework. By subtracting the coarser from the fine scales, the algorithm enhances small local regions that stand out from their surroundings. Hence, they attract our attention.

**Figure 4.**
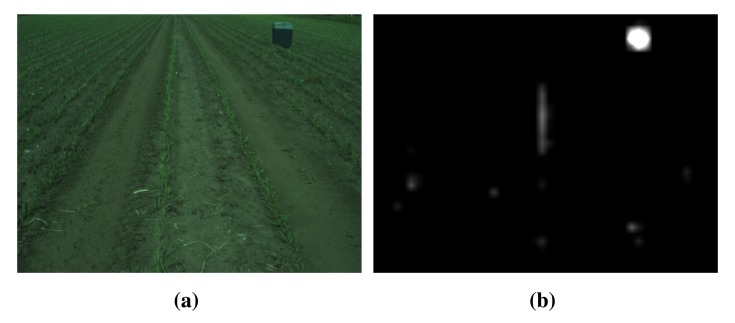
Example of visual saliency in captured data. (**a**) Input RGB color image; (**b**) resulting saliency map.

In Figure 4, an image from the field is shown together with the calculated saliency map. The saliency map is a gray scale image, where pixel intensity is related to how salient a region is. In the figure, it is seen that the cooler box in to top right corner is most salient, which makes sense, as it stands out from its surroundings.

In this research, we have utilized the saliency toolbox [23] for MATLAB to implement visual saliency. A more detailed description of the algorithm can be found in [16,23].

### 2.3. Background Modeling

In [9], a background model is based on an appearance model. This model assumes that obstacles differ in appearance from the ground, which is assumed to be relatively flat (this is utilized to calculate distance to the object). The background is based on a reference region, which is defined as a trapezoidal area in front of the camera. In [13], the same approach is used to model the background and adds tracking of corner features to navigate a mobile robot. In [9], both a simplified and an incremental version of the background model are presented. The simplified model is constructed based on the first captured image. The model is constructed as follows:

1. Smooth the input color image with the Gaussian filter;
2. Transform the image into the HSI color space;
3. Calculate the histogram of the reference region (the trapezoidal area).

In the presented algorithm, a pixel is classified as an obstacle if: (1) the hue histogram bin value at the pixel's hue value is below a hue threshold; or (2) if the intensity bin value at the pixel's intensity value is below an intensity threshold. As the overall appearance of the input images may change over time as the machine or vehicle moves, an update scheme of the reference histograms is presented in [9]. Here, the robot is manually steered through the environment, whilst avoiding obstacles, and histograms from regions without obstacles are used to update the model based on a simple OR function.

**Figure 5.**
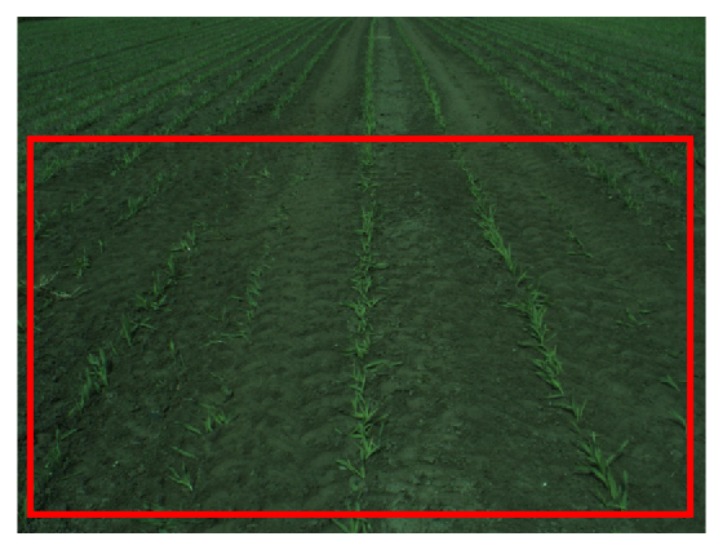
Initial reference region selection.

**Figure 6.**
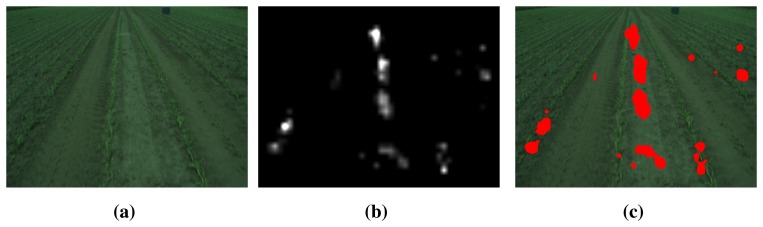
Example of visual saliency in captured data. (**a**) Input RGB color image; (**b**) saliency map; (**c**) red regions are not selected for the background model.

Like the algorithms described in [9,13], we assume that the background may be represented by an appearance model. This is a strong assumption for an outdoor environment; however, it exploits the homogeneity of a row crop field. The background model is constructed as follows:

1. Transform the image into the HSI color space;
2. Calculate the histogram of the reference region;
  (a) In the first image, a specified region close to the camera is selected as the reference region (Figure 5);
  (b) In the subsequent frames, the histograms are updated via an incremental update scheme based on input from visual saliency estimation (Figure 6).

In the initial frame, the reference region is defined as a specified region close to the camera, comparable to the methods in [9,13]. In Figure 5, the initial reference region is shown. This region is used to calculate the histograms for the background model.

In the subsequent frames, the reference histograms are incrementally updated based on saliency-based reference region selection. In the saliency-based selection, it is assumed that salient regions in the image are less likely to be part of the background. This assumption is also presented and utilized in [18,19], where salient regions are excluded from the ground estimation algorithm, as these regions are less likely to be part of the ground plane. In [21], saliency is utilized for background subtraction in highly dynamical scenes, with the underlying assumption that salient regions are part of the foreground. In Figure 6, the two stages of the reference region selection are shown.

The histograms of non-salient regions are utilized to update the reference histograms (denoted as *uHist*) in the following manner:

(1) $${\text{uHist}}_{t}[k]=\alpha \cdot {\text{Hist}}_{t}[k]+\beta \cdot {\text{Hist}}_{t-1}[k]$$

Here, *α* + *β* = 1 and *k* indicate the *k*-th bin at time *t*. After each update, the histogram are normalized, as the number of pixels used to calculate the histogram differs from frame to frame. In our algorithm, a pixel that is not part of the background is initially labeled as a foreground pixel, based on the threshold method described in [9]. These pixels are re-labeled as an obstacle (nest) or non-obstacle during fusion with the saliency map.

### 2.4. Fusion

**Figure 7.**
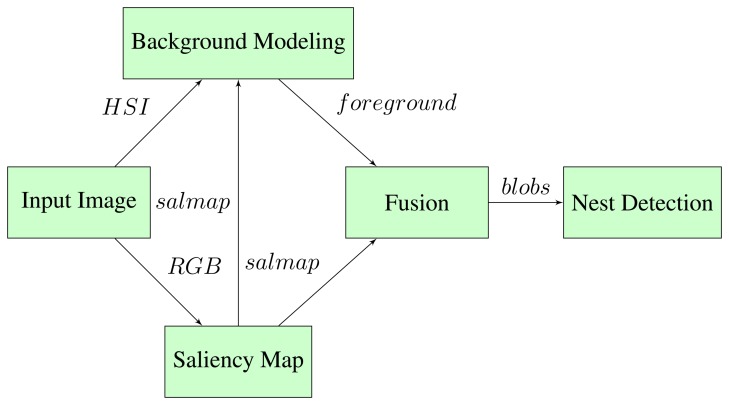
Flow of the nest detection algorithm.

In Figure 7, the flow of the nest detection algorithm is shown. For each input image, the saliency map (salmap) and foreground is found based on the algorithms presented in the previous sections. The foreground is a binary image, where a pixel value of 1 corresponds to a foreground pixel. The saliency map is a gray scale image, where pixel intensity is related to how salient a region is. The background modeling utilizes the saliency map to construct reference region histograms for each frame, as described in Section 2.3.

In the fusion stage of the algorithm, the most salient region is selected. This is implemented by selecting the salient region with the highest pixel intensity values. The saliency map is converted to a binary image, where only the pixels within the most salient regions has a value of 1. The foreground and the binary saliency map is fused by an AND operation. To remove noise, blobs smaller than a given lower threshold are removed, and the remaining blobs are detected. The output of the algorithm is the (x, y) position of the largest blob, if it is above a given area threshold.

## 3. Results

This section presents the results of nest detection using the presented algorithm. The performance is evaluated by the detection capabilities, including the detection range, of the algorithm. To evaluate the performance, all frames in the dataset have been manually labeled. Here, both the bird nests and the cooling box (visible in most of the dataset) have been labeled. The cooling box labels are used to suppress the cooling box in the algorithm.

The visual saliency algorithm is computationally complex, but frame rates above 25 for 640×480 pixel images have been reported in the literature [24]. Thus, we have re-scaled the RGB images to a resolution of 640 × 480 pixels, as it is required that the algorithm runs at a sufficient frame rate to ensure timely detection of obstacles.

### 3.1. Background Model Update

**Figure 8.**
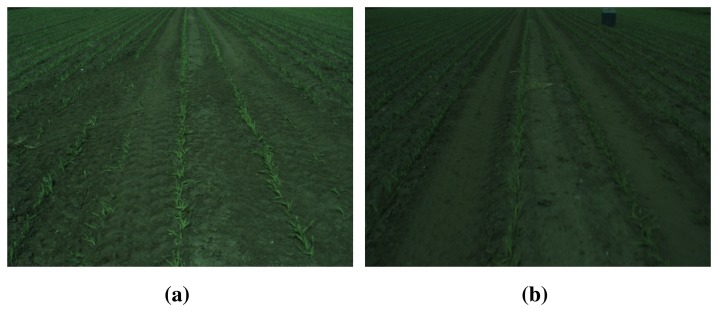

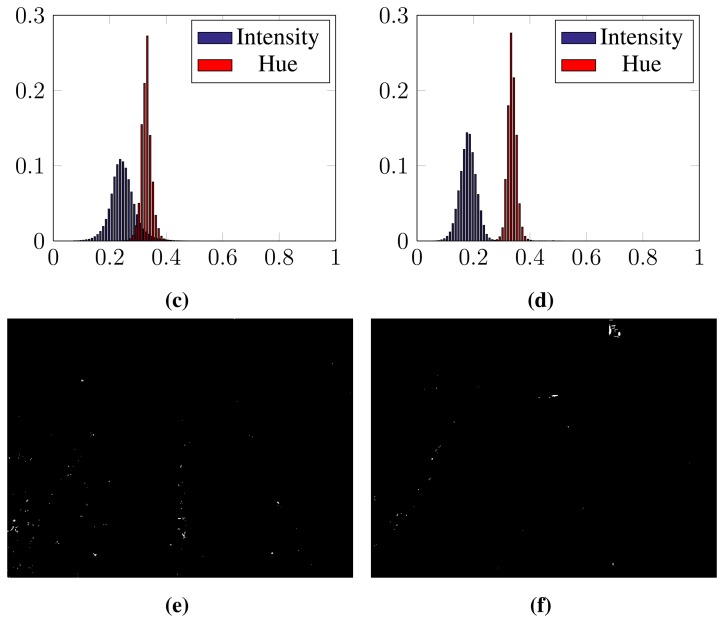
Incremental background model simulation. (**a**) Bright RGB color image (initial reference region); (**b**) dark RGB color image simulating a cloud; (**c**) background model for bright image; (**d**) updated model based on the incremental update of the histograms; (**e**) resulting foreground detection for the bright image; (**f**) foreground detection for the dark image (there is a nest just above the center of the image).

To investigate the performance of the incremental background model update, a change in image intensity has been implemented during algorithm evaluation. This change in intensity simulates a cloud covering the Sun for a few seconds, thus changing the intensity from bright to darker and back to bright again. In Figure 8, two images (Frames 1 and 40, respectively) from such a sequence are shown together with the background model, as well as the resulting foreground detection.

The intensity changes have been used in the following evaluation of the nest detection algorithm.

### 3.2. Nest Detection

In the evaluation of the algorithm, we used 0.00015 as a threshold for both the normalized hue and intensity background models. The lower area (for removing small blobs) was set to five pixels, and the area threshold for a blob to be detected was set to 30 pixels (after fusion with the saliency map). In Table 1, the results from the evaluation are shown in a confusion matrix. It is seen that the total numbers of false positives and true positives are almost similar. From the confusion matrix, the precision (PPV) and true positive rate (TPR) can be calculated.

(2) $$\text{PPV}=\frac{TP}{TP+FP}$$

(3) $$\text{TPR}=\frac{TP}{TP+FN}$$

Here, TPR = 0.38 and PPV = 0.53, respectively.

To maintain efficiency during weeding, the number of false positives needs to be decreased, as a false positive would require an avoidance action when it is not needed. Here, tracking can be used to improve the results shown in Table 1. It is also seen that the number of false negatives is high. This will be further addressed in the following text.

#### 3.2.1. Temporal Constraint

**Figure 9.**
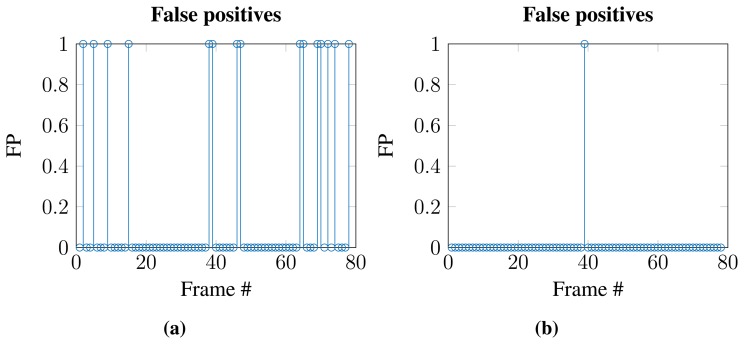
Reduction of false positives due to tracking. Here, three consecutive frames are evaluated. (**a**) False positives before tracking; (**b**) false positives after tracking.

An analysis of the temporal occurrence of, e.g., false positives in one of the datasets shows that many false positives can be avoided if a temporal constraint is applied (see Figure 9), meaning that the algorithm must detect the object in multiple consecutive frames. This has been implemented via a naive tracking scheme, where the position of the current detected object is compared to the position of the detected object in the previous frames. The newly detected object is the same for both frames, if the object has not moved too far, given by a distance threshold, in both the x- and y-direction [6]. If a detected object is not within the distance threshold, a new track is added. An object is identified as an obstacle (nest) if the distance threshold is met in two out of three consecutive frames. This ensures that detections, which only occur in single frames or at random positions, are not mistaken for a nest.

#### 3.2.2. Dealing with False Negatives

Due to the range of the system (1–15 m), the algorithm experiences many false negatives, as seen in Table 1. During the manual labeling, the positions of the nests were known; hence, the true positions of the nests could be labeled even if the nests were several meters away. In this case, the nests are very small, and the algorithm fails to detect them.

**Figure 10.**
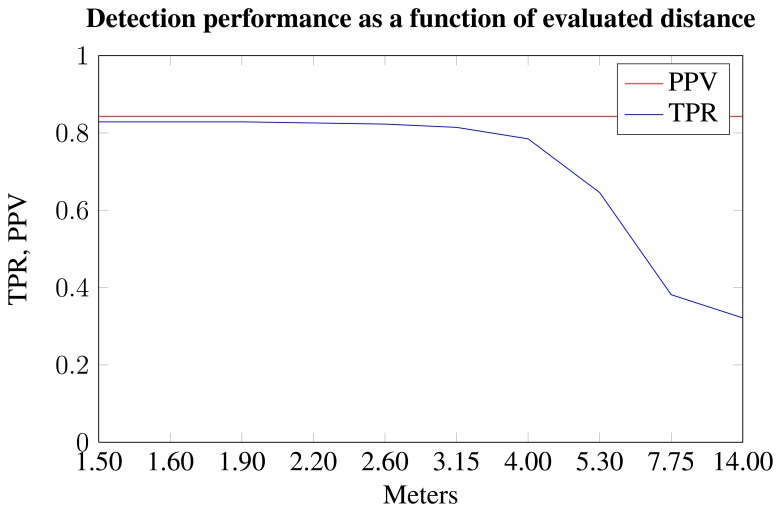
Performance as a function of evaluated distance.

In Figure 10, the TPR and PPV are shown as a function of the evaluation distance, e.g., 2.90 m means that the nest is only labeled within 2.90 m of the camera. This influences the number of false negatives, as seen by the decrease in TPR as the evaluation distance increases.

By implementing temporal constraints and limiting the evaluation distance, the algorithm achieves the performance shown in Table 2. Here, the evaluated distance has been adjusted to four meters. The algorithm was able to detect 28 of the 30 nests in the dataset, with a mean detection distance of 3.8 m. For these 28 nests, the detection performance is as shown in Table 2. The number of false positives and false negatives has been reduced compared to Table 1. The numbers in Table 2 gives a TPR of 0.75 and a PPV of 0.84.

## 4. Discussion

Automated nest detection may be an important tool for the improvement of wildlife-friendly farming practices and, as such, offers a potential for reducing wildlife mortality in agriculture. This is particularly the case for farmland passerines, many of which are of conservation concern.

The presented algorithm is generic and not limited to bird nest detection, as it detects obstacles based on background modeling and visual attention, rather than feature extraction and recognition. However, nests were chosen as the case study, due to the destruction of these nests during mechanical weeding and the, to our knowledge, non-existent research within automated nest detection for agricultural machinery.

There were two types of obstacles present in the recorded data: bird nests and a cooling box (used for egg storage). The algorithm is able to detect both types of obstacles, as it is generic, and finds objects that grab our attention. The algorithm detects the cooling box in all 15 recordings, with a TPR of 0.83 and a mean detection distance of 7.7 m. When detecting the cooling box, there are no false positives, as smaller obstacles can be removed due to size constraints. By relaxing the size constraint, the algorithm is also able to detect the smaller obstacles, such as bird nests. The performance of the bird nest detection is reported in this paper.

The nest detection algorithm achieves fair results, as it detects 28 of the 30 nests in the dataset, giving a detection rate of 0.93. The mean detection distance is approximately 3.8 m, with a true positive rate of 0.75. Hence, the algorithm is able to detect the nests in 75% of the frames in which they are present. The typical driving speed in current mechanical weeding is 10–12 km/h. Here, the the machine needs to be designed to react within 1.1 s given the achieved mean detection distance of 3.8 m. However, for autonomous mechanical weeding, the expected driving speed is around 5–7 km/h [25,26]. Here, the reaction time should be around 1.9 s, for the given distance, to avoid the nest. This puts some requirements on the mechanical design of the machinery, which should be designed to avoid the nests by raising the equipment from the ground, thus providing free space for the ground-lying nest. This mechanical operation could be performed much faster than trying to avoid the nest by driving around it.

The achieved detection distance could be improved by a higher resolution and increased frame rate. The recordings were performed at a low frame rate (5 fps), due to thermal camera synchronization (the thermal camera operated at this low frame rate). An increased frame rate could increase the detection distance, as the temporal constraint could be achieved over a shorter time span, thus increasing the detection distance capabilities.

An increased resolution would also increase performance, as the nest would be visible at an earlier stage. Increasing the resolution, however, is not free, as it increases the number of computations. Real-time implementations of visual saliency do exist in the literature [24], where the saliency of a 640 × 480 pixel image is calculated at almost 28 fps. However, at driving speeds of 5–7 km/h, the required frame rate is lower. We are currently implementing the algorithm in Python, where it runs at 15 fps at a resolution of 640 × 480, using Itti's algorithm for saliency [16]. The resolution could be increased by porting some of the algorithm to a GPU or by only calculating saliency in regions of the image that are detected as foreground. In this case, histogram-based saliency could be used for this [27].

The algorithm is designed to detect obstacles, big and small. In the case of nest detection, the detection distance and subsequent reaction time are lower than for larger obstacles, such as the cooling box. However, the action to be performed when detecting small obstacles is not to navigate around them, but rather to lift the part of the machine in danger of hitting the nest. This can be done much faster than navigating around it; thus, the requirements are lower. At a moving speed of around 7 km/h (2 m/s), the worst case is when the nests are detected at the bottom of the field of view. Given the setup in the experiment, this is one meter in front of the vehicle. This results in 0.5 s to react, which is sufficient for lifting the equipment. Given a frame rate of 15 fps and a temporal constraint of three frames, the detection distance needs to be 1.1 m in this worst case scenario. The achieved mean detection distance is 3.8 m, with the minimum achieved detection distance being two meters. Furthermore, the TPR is above 0.8 within these distances (see Figure 10); hence, the detection is more reliable. Thus, the algorithm is able to detect the bird nests in time, when driving speeds are equal to expected driving speeds for autonomous mechanical weeding.

The dataset was recorded over the course of one day. The weather was warm during the recordings, resulting in unusable thermal images. It is clear that the thermal camera is not useful for all scenarios, as shown in the dataset in this paper; however, for other, not so warm days, the thermal images could potentially increase performance, as nest temperature could be utilized for detection, as well [28]. Therefore, future work should include real-time implementation and fusion with thermal imaging to increase performance.

**Figure 11.**
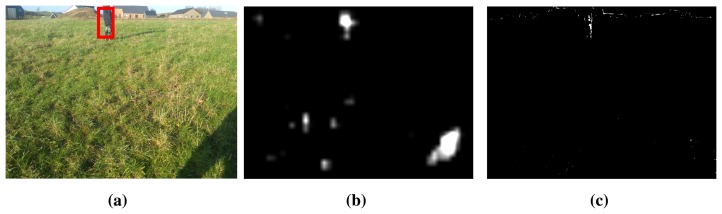
Example of the detection performance with shadows present in the image. (**a**) Detection of a child using the presented algorithm; (**b**) saliency map; (**c**) foreground pixels based on the incremental background model.

In the Results Section, clouds covering the Sun are simulated by decreasing and, subsequently, increasing the intensity of the entire image. The incremental background modeling ensures that the background model adapts to these changes. Another scenario is the presence of shadows, as in Figure 11a, where the shadow from the vehicle is seen in the bottom right corner. Here, the saliency algorithm highlights this region (Figure 11b). However, as the the ground is homogeneous in appearance, the background model ensures that the ground in the shadow is not labeled as foreground. This results in correct detection of the child and no detection of the shadow.

To maintain efficiency, different avoidance strategies for different types of obstacles are preferable. Hence, the type of obstacle needs to be recognized, which is a difficult task. Current machinery is able to avoid obstacles by raising the implement, driving around the obstacle or stopping. Therefore, we suggest that obstacle classification be based on the height of the obstacle, as this could provide useful information for the choice of strategy.

## 5. Conclusions

An algorithm for automated detection of bird nests in row crops has been presented. The algorithm is based on visual saliency and incremental background modeling. The incremental background model exploits the homogeneity of the field and thereby represents the overall appearance of the field.

Fusion between saliency maps and foreground detection results in the detection of ground-lying bird nests. The algorithm achieves fair results, as it detects 28 of the 30 nests present in the recordings, at an average distance of 3.8 m.

## Figures and Tables

**Table 1.** Confusion matrix of nest detection.

		**Predicted**	
		*Positive*	*Negative*
**Observed**	*Positive*	284 (TP)	453 (FN)
	*Negative*	245 (FP)	333 (TN)

**Table 2.** Confusion matrix of nest detection after tracking and limited evaluation distance (four meters).

		**Predicted**	
		*Positive*	*Negative*
**Observed**	*Positive*	237	78
	*Negative*	44	956
